# Exploring the Needs, Expectations, and Realities of Mental Healthcare for Transgender Adults: A Grounded Theory Study on Experiences in Sweden

**DOI:** 10.1089/trgh.2017.0033

**Published:** 2018-05-01

**Authors:** Debra Carroll-Beight, Markus Larsson

**Affiliations:** ^1^ Faculty of Medicine, Lund University, Malmö, Sweden.; ^2^ Division for Social Medicine and Global Health, Department of Clinical Sciences, Malmö, Lund University, Malmö, Sweden.

**Keywords:** constructivist grounded theory, qualitative methods, mental healthcare, Sweden, transgender, transition-related care

## Abstract

**Purpose:** Transgender persons experience a disproportionate representation in adverse mental health conditions globally. In Sweden, there are tangible efforts to improve mental healthcare overall, but transgender persons still struggle with meeting their mental healthcare needs and there is an absence of understanding the role of mental healthcare for this population and how services are being utilized. Thus, the aim of this study was to gain knowledge from transgender individuals in Sweden concerning their mental healthcare, their needs, expectations, and realities, regardless of transition status.

**Methods:** Eleven in-depth interviews were conducted with persons who identified as transgender, older than 18 years, at some stage of transition in Sweden. Data were collected, analyzed, and interpreted using constructivist ground theory.

**Results:** Three categories emerged from the analysis, Feeling Objectification Rather than Subjectivity, Constructing the Narrative, and Reflecting on Aspects of Care that illustrate the dual tensions at play in transgender visibility, communication with mental healthcare professionals, and expectations of care. Six subcategories further delineate the specific forces at work that construct the mental healthcare experiences for trans persons.

**Conclusion:** Increased knowledge and visibility of transgender persons are needed to adequately serve the mental healthcare needs for this population. Currently, there are barriers that inhibit transgender persons from getting the mental healthcare assistance desired and needed, as they do not view the healthcare system as safe space. As steps are being taken to depathologize transgender identities, momentum should be continued to create space for trans persons that enables unencumbered mental health assistance.

## Introduction

The World Health Organization (WHO) defines mental health as, “a state of well-being in which the individual realizes his or her own abilities, can cope with the normal stresses of life, can work productively and fruitfully, and is able to make a contribution to his or her community.”^1^ However, it is typically within the deficiencies and obstacles where public health actors must address health to move individuals and communities toward a holistic well-being.^2^

For certain vulnerable individuals and groups, there is a higher risk of experiencing adverse mental health. These include those living in lower socioeconomic circumstances, ethnic or racial minorities, and those who experience stigma and discrimination due to their sexual orientation and gender identity such as lesbian, gay, bisexual, and transgender (LGBT) persons.^1^

From a biological perspective, transgender persons experience mental health issues in the same ways as cisgender persons. This means that being transgender does not predispose one to be at a higher risk for disorders.^3^ However, transgender individuals do suffer from higher incidences of mental health illnesses such as depression, anxiety, and suicidality often stemming from the social implications of being transgender in a cisnormative society.^4^ Subsequently, resulting mental health issues, resiliency, and quality of life are impacted in ways that are again similar to effects on cisgender persons but still are contentious and amplified for marginalized populations.

There is currently a relatively rich body of literature exploring the mental health among transgender populations.^3,5–9^ Reisner et al.^9^ asserted in the Lancet series on transgender global health that data “consistently showed that transgender adults are burdened by mental health concerns.” In the United States, a recent survey showed that transgender persons reported high rates of psychological distress issues such as “depression, anxiety, and suicidality at 39% compared with 5% for the general U.S. population; 40% had attempted suicide at some time in their life compared with 4.6% in the general population.”^4^ Similarly, a study from 2015 in Australia indicated that 46% of transgender persons reported high to very high levels of psychological distress over the course of the previous year.^7^ Researchers in Mexico attributed an underlying force for the high rates of mental health issues in this population that arose from a response to “discrimination, stigma, lack of acceptance, and abuse” experienced on a regular basis and not from inherent biological factors.^3^

In 2015, the Swedish Public Health Agency conducted the largest public health survey aimed at the transgender population, surveying 800 transgender persons from across Sweden.^10^ Regarding mental health, results showed that 36% of respondents had contemplated suicide at least once during the previous year, six times higher than the national average.^10^ Five percent had attempted suicide at least one time in the previous year, five times higher than the national average, and 1/3 had attempted suicide at some point during their lifetime.^10^ Furthermore, 72% of respondents who had attempted or contemplated suicide indicated that a portion of their trans experience was the cause for their intentions but not the sole defining factor.^10^

In another study from Sweden that assessed the quality of life reported by transgender individuals, 44% recorded a low quality of life with 73% noting a lack of medical support as a driving force of this low quality.^11^ Looking at the previous studies on transgender persons and mental health concerns, there is a notable lack of transgender voices, specifically relaying their needs and expectations for mental health support that were unrelated to transition care. It is within this absence that this study seeks to highlight the voice of transgender individuals in Sweden to better understand what influences their experiences with mental healthcare in this setting and the ways in which transgender persons navigate these influences. Thus, the aim for this study was to gain knowledge from transgender individuals concerning their mental healthcare, their needs, expectations, and realities, regardless of transition status. The theory of subalternity, which deals with the authoritarian representation of groups and individuals in hegemonic systems, is used to highlight how hegemonic views of trans persons as specific subjects with specific identities affect their abilities to maintain their mental health. This knowledge is needed to identify strengths and weaknesses in transgender healthcare and to identify ways to best serve this population to meet the health needs.

## Methods

Before commencement of this study, a literature review was conducted on transgender persons, transition-related healthcare, and mental healthcare. The gathered knowledge helped developing guiding research questions, based on current knowledge gaps and needs, which in turn were instrumental in refining the aim to gain knowledge from transgender individuals concerning their mental healthcare, their needs, expectations, and realities, regardless of transition status.

To understand the subjective nature surrounding the social processes that affect perceptions experienced by transgender persons, a qualitative approach using a constructivist grounded theory based on Kathy Charmaz was used.^12^ Charmaz's approach works well in that it focuses on giving voice to those directly involved, highlighting their lived experiences.^12^ In utilizing this method of grounded theory, the relationships that exist between transgender persons and their experiences with mental health and mental healthcare can be illuminated.^13^

This constructivist perspective treats “the research process itself as a social construction” in recognizing that meaning is a cocreation between the researcher and informants, which guides the decisions and directions undertaken as well as the analysis process.^14^ This cocreation principle emphasizes the obligations of the researcher to think through actions and choices, maintain critical reflexivity, and scrutinize each step in the process.^14^ To guide this, the authors reflected on philosophical assumptions related to qualitative inquiry applying Charmaz's lens to this approach. This defines the relativist ontological position as one that to remain true to the transgender persons' experiences, we must adopt their perspective lens of reality and use their words as sufficient evidence.^13^ Reflecting on the epistemological stance of constructivism, the author acknowledges the collaborative interrelationship between the researcher and informants in discovering and identifying meanings in experiences.^13^ To address the axiological implications of this study, the authors evaluated value biases and preconceptions through extensive reflexivity notes during all phases of this study. The purpose of this is to promote transparency and assist in validity by proactively limiting the influence of bias.^12^ Finally, being mindful of the methodological process, specifically with the logical progression of analysis, and by keeping the context in focus throughout the study. Emergent inquiry developed through working with details before generalizations, systematically looking at the data in the development of categories.^15^

### Study setting

For mental healthcare, Sweden has demonstrated a commitment to improved healthcare services in terms of public policies, legal protections, and resource allocation.^16^ However, there is still a gap for certain populations, specifically minority populations such as transgender individuals, as seen in the 2015 Public Health Agency Report, and thus indicates a need for “adapted outreach efforts,” specifically addressing this populations' treatment needs.^16^

To address the current needs of transgender persons in Sweden, there are six Gender Identity Assessment Teams (GIATs) servicing transition procedures. These teams operate under the National Board of Health and Welfare, which coordinates and regulates care services and costs between the central government of Sweden and the city councils and municipalities.^17–19^ Each team, working with its regional board, has varying regulations regarding referrals for treatment. However, most GIATs require a psychiatric referral to begin the process, while all GIATs require psychological and psychiatric assessments to obtain treatments.^17^ This study sampled persons who work or have worked with three GIATs, located in the three largest urban regions of Sweden (i.e., Stockholm, Gothenburg, and Malmö/Lund).

### Recruitment process

Initial recruitment began by advertising through Lund University's student-run sexual health group, Projekt Sex (P6), reaching out to members with a Facebook post and e-mail explaining the study purpose and providing contact details to the author asking for referrals. A similar message was also sent to three membership organizations working with LGBT, via their e-mail addresses provided on their websites. Members shared this introductory message to closed transgender groups on Facebook for persons in Malmö and Göteborg. Once interested persons initiated contact with the first author (D.B.), a detailed letter explaining the study was sent via e-mail or Facebook Messenger. Study participation was open to persons 18 years or older who identified as transgender or nonbinary at any stage of transition, contemplating, initiating assessment, midprocess, or completion. Persons who wanted to continue were scheduled for an interview (see Appendix 1). Although several persons from Göteborg and Stockholm expressed interest in participating, constraints on time or travel issues did not allow for all of those potential interviews to commence. Participants were seven persons from the Lund/Malmö area, one person from Göteborg, and one person from Stockholm representing the three GIAT catchment areas for the three larger cities in Sweden. A pilot study with two transgender persons from Malmö was conducted in May 2016. This previous study helped with the emergent design in shaping the final aim, research questions, and interview guide, and furthered the analysis of mental healthcare in relation to transgender persons. Interviews commenced from February through March 2017 by the first author. In total 11 participants were recruited.

Before each interview, participants were e-mailed a copy of the study information letter explaining the details. Ten interviews were face-to-face, and one was via a video/audio-based communication app due to travel constraints. On meeting for the interviews, participants were offered a physical copy of the information letter, the letter was reviewed to highlight the voluntary nature, ability to bypass any questions or topics or to withdraw altogether, accommodations for anonymity and confidentiality, and explanation of the recording procedure and measures to ensure data protection and subsequent transcribing of the interview. Participants were asked if they had any other questions or concerns to address before proceeding and then each was asked to sign a consent form.^*^ Interviews were recorded on an electronic tablet with recording capabilities with a password-protected program. For the face-to-face interviews, participants were invited to select locations of preference to ensure comfort and to their level of discretion for privacy. Refreshments were offered as appropriate, but no other compensation was given for participants. Interviews ranged from 40 to 90 min.

Data were collected through in-depth, semistructured interviews. Questions were thematically structured in an interview guide (see Appendix 2) to cover a range of areas, including general thoughts on mental health and mental healthcare, mental health relating to transition, mental health unrelated to transition, and finally expectations of care. Directly following each interview, ∼1 h was dedicated to writing memos and reflexivity notes. Substantive memos detailed setting and participant descriptions as well as conversational quotes, and methodological memos covered author comments, observations, and reflections of successful elements of each interview and areas that needed adjustment or improvement.^15^ Analytical memos served as the foundation for analysis of the data, summarizing ideas and themes that emerged from the interviews that could be developed into codes and categories.^15^ Memoing continued throughout the study analysis allowing the author to reflect on the data in concrete and abstract portions, permitting the emergence of connections between codes and ultimately theory formation.^15^ Interviews were transcribed verbatim and entered into open code to commence analysis.^20^

### Data analysis

While using a grounded theory approach, interviews were transcribed and initial coding and memoing occurred simultaneously with data collection.^15^ Using Charmaz's framework, the analysis was performed in sequential steps from initial coding to focused coding and to conceptualizing theoretical codes that eventually defined the sub- and main categories, all while integrating the simultaneously recorded memos.^12^ Relevant ideas were classified and developed, resulting in 249 initial codes. The next step was to continue to refer back to the text, comparing the initial codes, merging similar codes, and becoming more selective and conceptual to cultivate the analytical direction of the focused codes. Memos were integrated into the analysis and codes and concepts were refined to clarify relationships. This continued for the interpretive understanding that defined explicit and implicit connections that raised focused codes to the conceptual theoretical codes represented in the subcategories and categories (see Table 1).^12^ The analysis was conducted by the first author (D.C.-B.) performing the initial coding and then conferring and finalizing with the second author (M.L.) to reach consensus.

**Table 1.** **Text to Category Example: Feeling Objectification Rather Than Subjectivity**

Interview text	Initial code	Memo	Focused code	Concepts/theoretical codes	Subcategory
“I think my gender reassignment procedure, changing my appearance, will affect me in unique ways. But these are issues that are highly connected in the gaze of transgender persons but it is not something that we are talking about”	Gaze of transgender persons	Altering the idea of the “male gaze” to a trans perspective. It's still an external view that objectifies. Not being talked about	Being Seen	Appearance	Being Seen
				External view	
				Ways of looking at	
				Denying subjectivity	
				Commodifying	
				Degrading	
				Denying autonomy	
				Ownership	
				Silencing	
“So okay if it means non-binary are getting treatment…I think its positive. But really I wish it wasn't so much about the body itself it was more about how you choose to present yourself no matter and that you are”	Not being all about the body	Shifting focus from the body to presentation. Are these disconnected? Who makes the decision if it's “about the body”	Not focusing on physicality	Appearance	See Me!
				Internal and external perceptions	
				Wanting to be seen	
				Personal	
				Contextual	
				Connect to subjectivity	
				Intelligibility	
				Pushing against expectations	
				Creating new spaces to be seen in	
				Directing	
				Deciding	
				Resistance	
“I would like to choose clothing and things that didn't depend on seeing me as a male person but I still do. I also have personal taste of course but my personal taste is separate from gender norms”	Separate from gender norms	Resisting gender norms and being accepted/seen as an individual. Not being dependent on how others view me	Not being reduced to expectations		

### Ethical considerations

Although ethical approval was not sought out from the review board, ethical considerations for this study were in line with the principles of the Declaration of Helsinki.^15,21,22^ Written consent was obtained, confidentiality was assured through assigning code numbers instead of names, and data storage was password protected. The purpose of the study was clearly explained both in writing and reiterated verbally to all participants. Participants were told of their right to withdraw at any time without consequence and their ability to ask questions at any point of the study, including after participating in the interview. Permission was also taken for recording the interview. While participation was not expected to cause harm to participants, referral information for LGBTQ friendly counseling and mental healthcare services was made available to participants if they felt that they needed to address any issues that arose during the course of participation or thereafter.

## Results

Participants in this study included 11 persons, ranging in age from 18 to 64 years at various stages of transition, including pretransition (formal process with the GIAT had just started) through completion (all desired procedures were complete at the time of the interview). Participants had had less than a full year with formal transition through completing transition 24 years ago. Six participants had been assigned male at birth and five had been assigned female at birth. Ten participants actively sought out mental healthcare services that were unrelated or unconnected to their transition process (see Table 2 for characteristics of participants).

**Table 2.** **Characteristics of Participants**

Participant	Country of birth^a^	Age at time of interview [M: 32.09 SD: 14.67]	Age at initiation of formal transition	Assigned sex at birth	Self-identified gender	Transition status^b^	Sought additional/external mental healthcare
1	Sweden	18	18	Female	Male	Beginning (<1 year)	Yes
2	Sweden	24	18	Male	Woman	Completed (6 years)	Yes
3	Sweden	32	25	Female	Trans person/trans guy	Completed (4 years)	Yes
4	United States	35	18	Female	Trans guy with trans feminine tendencies	Completed (9 years)	Yes
5	Sweden	64	30	Male	Female	Completed (10 years)	Yes^c^
6	Germany	56	55	Male	Trans sexual female	Beginning (<1 year)	Yes^c^
7	Sweden	23	20	Male	Trans woman	In process (3 years)	Yes
8	Netherlands	29	22	Female	Man, trans man, nonbinary, demi-guy, depends on space	In process (7 years)	Yes
9	Serbia	25	25	Male	Transgender woman	Beginning (<1 year)	No
10	Sweden	23	19	Female	Man	In process (4 years)	Yes
11	Sweden	24	18	Male	Trans woman	In process (6 years)	Yes

^a^ All participants, regardless of country of birth, were eligible for state-funded healthcare under the Swedish system.

^b^ Not accounting for interruptions in treatment.

^c^ Informants sought informal counseling rather than psychological or psychiatric care services.

M, mean; SD, standard deviation.

The results showed that participants grappled with tensions created through opposing influences, affecting their ability and inclination to address their mental health needs. These stemmed from internal and external sources that constructed thoughts on gender as well as mental health. Subsequently, this influenced the ways that trans persons navigated systems designed to assess and treat their gender identity and mental health needs. Illustrating the interconnections of these views and processes, three categories emerged that presented an element of duality that exists in these tensions. The three categories describing these experiences are *Feeling Objectification Rather than Subjectivity*, *Constructing the Narrative*, and *Reflecting on Aspects of Care*. Within these three main categories are six subcategories that work in tandem and further define the interplay of competing forces that create tension within transgender individuals, trans healthcare, mental healthcare, and society at large. The findings are presented below with main categories listed in bold and subcategories listed in italics and underlined.

### Feeling objectification rather than subjectivity

The category of *Feeling Objectification Rather than Subjectivity* demonstrates the external perceptions of transgender persons and their needs versus their internalized experiences. The two subcategories of *Being Seen* and *See Me!* illustrate the push and pull in this dynamic expressed by transgender individuals. Participants reported that they were simultaneously viewed as an object by medicine and society while existing as a subject of lived experiences. The tension exists as being seen as an object of evaluation. This evaluation constructs how care decisions are made or not made and was seen as being at odds with the needs of trans individuals.

#### Being seen

Participants noted feelings of being seen through narrow views that adhered strictly to constructed gender binaries and felt they limited the contextual understanding of their experiences. A general sense of objectification was perceived in addressing the need for obtaining a diagnosis for transition.

They only have narrow visions of gender and how you should be and act and what to wear. Saying [to me], ‘you're looking kinda manly, you have a blue shirt on, yeah it kinda suits you’….and then asking what I had on under, it was humiliating. (Participant 3)

This was extended in interactions with other mental healthcare services as the state of being transgender was perceived as distracting providers. Participants felt a further objectification in the need to confirm their identity rather than addressing mental health issues.

I want them to know where I need help. And then being trans comes second to that. I don't need help coming to terms with being trans, I'm already there, we can skip that part! What I'm struggling with now is that my brain keeps telling me that I want to die and on a rational level I know I don't want to die so can we do something about that? (Participant 8)

An aspect of *Being Seen* was that to convince healthcare professionals of the urgency felt by trans persons to address their transition or mental health concerns, a specific gender performance was required. This need to enact a specified performance was manifested through participants acting out particular masculine or feminine gender tropes, viewing these performances as vital to their survival in cisnormative systems. For the GIATs, they needed to present their dysphoria as something bad but not too bad, meaning that their mental health could not be called into question to a point where it would delay transition.

I tried to look and act as a stereotypical girl for them, as best I could at the time. It's walking a fine line. You have to feel very bad with your body and all but you can't be in too bad condition then you won't get any help so you have to balance. (Participant 2)

For mental healthcare providers unrelated to transition, participants considered whether or not to discuss the transgender issue, specifically when they felt that being trans was unrelated to their mental healthcare needs. For them, being seen as trans was not always desirable.

Quite often when I visit the doctor I don't even want to mention that I'm transgender because it's not relevant and people know too little about it so it just confuses them…when I've been talking about stress, I think people miss the point and just want to talk about being transgender. (Participant 2)

#### See me!

Being trans is who you are. (Participant 1)

For participants, the desire for recognition of one's identity was highly present. It speaks to the privilege of defining oneself rather than relying on others to do it. Participants expressed a frustration that they were not being listened to or their concerns were not taken at face value by the healthcare providers with whom they interacted. Exasperated by the lack of communication felt from the GIAT, one participant forcefully demanded of the psychologist:

What do you see?!? And then she finally said, ‘I see [participant's name]’ and you know, I really needed that, that's the most important thing. See me! This is me, confirm me! (Participant 5)

“Confirmation” of gender was wanted as an acknowledgment of being seen as “normal” or in line with prevailing norms. This was poignant as participants remarked that norms are invisible so the idea of wanting to be seen as an individual was in tension with the desire of being invisible. Being invisible for them meant being a part of the unnoticed norms that exist for cisgender society and gives a sense of belonging and safety. Another example of this tension was expressed by comparing being transgender with being left-handed. That on an individual level the left-handedness must be acknowledged in that one is operating in a right-handed world, but this variation was not a cause for increased attention or focus.

I'm left handed so I would need help or different tools to do my job well…That wouldn't mean that I'm ill at all but only that I have this functional variation, I'm not so different from anyone else. (Participant 5)

### Constructing the narrative

The category of *Constructing the Narrative* reflects the stories that participants developed for interactions with their healthcare providers and how these stories were interpreted. The two subcategories of *Following the Script* and *Defiant Silence* illustrate the tensions between the specific scripts constructed by participants that were relayed to and often expected from the mental healthcare providers and the ways in which participants resisted this predefined narrative by not talking about issues as a means to reclaim agency.

#### Following the script

Participants often followed a stereotypical script in defining their transition and mental healthcare needs. This script has numerous influences but it resulted in an internalized and externalized narrative that permitted participants to get the care they needed. It also reinforced a single view of how trans persons are supposed to be.

I basically said what I knew they expected me to say just to get it over with. So I could get the diagnosis, so I could get the healthcare I needed to survive, I had no other options. So it was like taking a test, you have to get the answers right and you'll do anything to be right. (Participant 2)

This story construction framed the interactions as artificial rather than as an individualized process. While participants felt adherence to the “classic, true trans story” was necessary, they understood that it reinforced a standardized story that did not adequately account for the varied ways of being transgender.

There's been a such narrow idea of what it means to be trans so people end up in this situation where they've heard this kind of script that is approved and then they just basically feed that to whichever mental healthcare professional that they are dependent on for their approval. So like ‘yes, I was ripping the heads off my dolls when I was three and I wanted to play with trucks, and every time I was put into a dress I had a melt down and I shot my mother in the face’ (laughs). You get this classic, true trans story, like a checklist and it's unfortunately what happens when you have people in these positions of power and you figure out what works and what doesn't. (Participant 4)

#### Defiant silence

The feelings of objectification and narrow perceptions of trans persons led participants to be mindful of the stories they told mental healthcare providers. As a way to negotiate their own sense of agency, participants purposefully withheld thoughts and feelings as an act of self-defense. Seeing relationships with healthcare providers in almost adversarial terms, participants took back their power by directing what they would speak about and what they would not.

The major problem in terms of treatment of transgender persons is when those mental health problems I might identify with, when you articulate them, they are a kind of a barrier that stands in the way of treatment and it delays many things so that's why I think we are silent about anything occurring in our lives. (Participant 9)

As mentioned previously in the subcategories of *Being Seen* and *Following the Script*, participants felt the need to fit into the perception of a suitable trans candidate. Because they needed to be seen as mentally stable for the GIAT to be permitted to transition, participants would censor themselves to align with this perception.

I never lied to the doctors but there were subjects that I never discussed, because I knew they might not like it, so it wasn't like an open discussion, I wasn't open with them with all things, I didn't risk it. (Participant 2)

When speaking with mental healthcare professionals outside of transition, participants indicated they would downplay elements of being transgender, if they addressed it all, so that the focus did not center on them being trans rather than having a mental health concern.

I felt that dealing with or getting more of a hold with my depression will probably happen when we start this f***ing thing not just by talking more about me being trans! (Participant 4)

### Reflecting on aspects of care

The final category of *Reflecting on Aspects of Care* examined both the current attitudes toward mental healthcare that were related or unrelated to transition and the desired expectations for ideal care. The two subcategories of *Fearing the System* and *Wanting Care That Cannot Wait* present the tension that manifested in that there was a strong need for reaching out for care but a continued distrust in the systems and providers in place to give that care.

#### Fearing the system

While the process of gender assessment was often referred to as frustrating and drawn out, it was the fear of how the “system” worked that impacted how participants chose to deal or not deal with their mental health concerns. As mentioned previously, limitations on how trans persons felt they were viewed by mental healthcare professionals, related and unrelated to transition, impacted their perceptions of care. For participants, the system created feelings of dependence and obligation. For them, they were at the mercy of a system that decides life and death, sometimes figuratively, often times literally.

People are dying, dramatic as that sounds, its real…people like us are in pretty big danger of death either by suicide or anything else and it feels weird to be in this position where you have to say ‘I'm at the risk of death if I don't get help’ and yet that help [transition] may be denied. Most of my friends and I joke about how many suicide attempts or thoughts we've had (laughs) because there's no other way to freaking cope with it. (Participant 7)

A generalized sense of distrust and resentment was expressed by participants in how the process of mental healthcare was addressed, the inflexibility of procedures, and the burdens felt by participants to behave in particular ways.

If you're asking a trans person- listen to them…[regarding mental health concerns], this psychiatrist was interrupting me and saying ‘that's not really accurate’ but I was like ‘what do you know, you're not in the community, who are you to talk about it?’. I mean they don't really have the perspective of us, they don't really care. They want to be critical but they don't offer insight. (Participant 3)

A lack of communication was another area of concern for participants, who felt that they were asked to lay their lives out and received nothing in return but feelings of being judged.

You never had any feedback, nothing! You're there and you're like something to be studied and you don't know what they are thinking and then what will happen? Will they just dismiss me like I'm not relevant here or what? What will happen, you never knew, you were in the dark. (Participant 5)

Participants felt their motivation to ask for assistance was inhibited by their concerns of how the system viewed them, instead choosing to suffer with their depression, anxiety, or suicidal thoughts, rather than risk delaying transition or impacting their process in anyway. This fear of the system inevitably affected their day-to-day functioning as akin to feeling like a prison inmate.

Like someone sitting in prison, you'd ask for permission for everything and other people are choosing things for you…So when you get the chance to really choose for yourself, you keep on asking for permission, it's so much a part of you now. (Participant 3)

#### Wanting care that cannot wait

For envisioning ideal care, a prominent idea was about urgency. Delays, either through excessive waiting times or interruptions to treatment in transition, caused harm to the overall mental health of trans persons.

I think I would have eventually got the diagnosis but it would have been delayed or something and I was worried. They have so much power, they can decide whether you live or die but I don't think they see it that way…you don't have time to mess around, you just want to get it [the diagnosis] as soon as possible. (Participant 2)

Another aspect to creating “care that cannot wait” was to include trans persons who had already completed the transition process, as advocates or as mentors into the care plans. This was seen as foundational necessities to improve care overall, both in transition and for mental health assistance. Participants felt this could provide desired benefits for the overall mental healthcare. This additional support would involve addressing practical needs for trans persons such as needing assistance in navigating the coming out experience, or even with legal, or employment concerns relating to their trans status.

Having a patient perspective and incorporating mentors is a start…helping me with all of these practical things like trying to navigate the labyrinth that is the State. Having this person know what's going on and be like ‘hey you need to call this person and see this person and fill out this form and I can help you with that’. Practical stuff is really helpful. (Participant 4)

Finally, a separate, external care model was desired, one that incorporated persons with diverse knowledge of LGBT issues as well as persons with better insight into transgender concerns.

When it comes to mental health there is a problem that we as transgender persons meet professionals that are all cis people…they don't know how to relate to what's important for us. So they need more knowledge, I think there should be some trans persons in these care plans. (Participant 5)

## Discussion

The aim of this research was to gain an in-depth knowledge from transgender individuals in Sweden concerning their mental healthcare, their needs, expectations, and realities, regardless of transition status. The findings of this study provide unique insights into the challenges that trans persons face with their mental healthcare and the strategies used to address their mental health concerns. By centering on the viewpoint of transgender persons, this research sought to increase their visibility and gain awareness of their lived experiences in an area that is still relatively undersurveyed.

### Conceptual model: the mirror

**FIG. 1.**
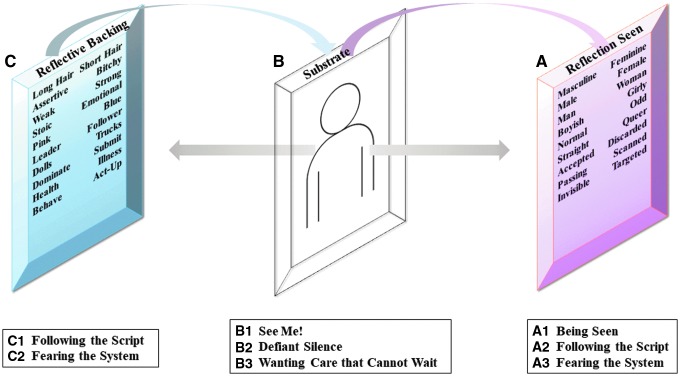
Conceptual model of subcategories—the construction of the mirror. The representation of interconnected categories that emerged from the analysis is indicated **(A)**, **(B)**, and **(C)** in the model and the respective subcategories **(A1–A3)**, **(B1–B3)**, and **(C1, C2)**. **(A)** The reflection seen is the image projected from the reflective backing placed on the mirror's substrate. It represents the social and cultural perspectives of how transgender persons are viewed externally and internally. **(B)** The substrate is the physical foundation that holds the backing and reflects out the image. This represents the construction of embodied subjectivity of trans people, the push and pull of norms, and how trans persons challenge these norms. **(C)** The reflective backing is the reflective coating placed on the substrate that creates an image. This represents the constructed social and institutional processes that influence transgender behavior and presentation and like the reflection seen is both internally and externally experienced.

To understand these findings and how they relate to trans experiences with mental healthcare, it is useful to present them in a conceptual model (see Fig. 1). This model depicts the construction of a mirror and represents the layered, interconnected, and sometimes competing forces that affect how trans individuals received care and their experiences of handling mental health issues. As the categories are interwoven and reflective of one another and not clear divisions of the forces at work, the subcategories are displayed to illustrate the nuance between influences.

The foundational substrate for the mirror represents the essence of the transgender persons and the ways they attempt to construct their own intelligibility. The reflective coating applied to the back of the mirror that creates the image to be seen is the social constructions that develop transgender performance, narratives, and perceptions of identity. It speaks to both the external and internal influences over the formation of transgender identities. The reflection seen from the mirror illustrates the ways that transgender persons are viewed externally by healthcare providers and by extension, society in general as well as the reflections they see of themselves. Underpinning these processes at work in the conceptual mirror model is the theory of subalternity, with interpretations by Antonio Gramsci and Gayatri Spivak.

### Gramsci and Spivak: subalternity

The interpretation of the subaltern from the Marxist political activist Gramsci focused on the social suffering of marginalized groups under hegemonic dominance of the elite class.^23^ Gramsci et al.^24^ described subalternity as the condition under which underrepresented persons or groups were subjected to the hegemonic culture and the activities that create invisible norms and practices of the dominant group. Spivak's deconstructionist perspective of subalternity is also relevant to interpret the study findings as it sheds light on how institutional structures fortify the hegemonic dominance, and the way this influences how the subalterns sees themselves within these systems.^25^ Instead of looking at only the cultural hegemonic processes, Spivak shifts focus onto the institutionalism of practices as a means of reinforcing norms and how this filters down into the behaviors of the subalterns so it works in tandem with cultural norms.^25^

### Reflection seen from the mirror

The reflection seen (Fig. 1A) illustrates the perceptions, assumptions, and evaluations made regarding transgender identity, presentation, and performance and how these form viewpoints about transgender persons, their mental healthcare needs, and the ways care is provided to them.

The subcategories of *Being Seen*, *Following the Script*, and *Fearing the System* (Fig. 1-A1 to -A3) encompass the social processes that impact the subaltern status of transgender persons. The hegemonic perspectives, exerted in the interaction between providers and patients, viewed the trans individual as an object for analysis, devoid of subjectivity with an expectation of specific performances. Participants were at the mercy of social influences that placed the obligation of normality on the individual to adhere to or otherwise risk continued marginalization. This finding is supported in a recent U.S. study on the impact of hetero-cisnormative attitudes on explicit and implicit transphobia.^26^ The findings suggest that the focus on gender norms affects perceptions of normality toward individuals, objectifying the individual and reducing them to a series of acceptable or unacceptable gender practices.^26^

#### Being seen

*Being Seen* (Fig. 1-A1) relates to being studied for determination of specific gendered knowledge from a position of hegemonic objectification. Feeling as they are seen only through a binary lens, transgender persons lose the contextual framework of their lived experiences and are limited to a predetermined mold of what it means to be trans. So, for example, participants in this study referred to the burden of “others” making choices for them based on how well or how poorly they presented their identity. Being objectified for study, they felt pressure to prove they were trans enough to receive transition support and then still felt objectified as being only trans when seeking external mental healthcare. The perceived need to pass and maintain a level of invisibility as a means to protect one's identity and personhood is reflected in numerous studies on transgender identity development, coping, and basic survival motivators.^27–31^

#### Following the script

The findings of *Following the Script* (Fig. 1-A2) connect to the meanings and “truths” formulated to determine what it means to be acceptably trans and how participants navigated care by following the “classic trans story,” to obtain treatment. This could be related with what Gramsci writes regarding constructions of the subaltern and how these images wrestle with meanings and the accepted norms and truths held throughout social relationships.^32^ For participants in this study, fulfilling a checklist to receive transition care was their primary focus even though it continued to solidify the accepted “truths” of what it meant to be trans, whether these were true or not. This skewed the perceptions for participants in their understanding of what care is supposed to mean, which consequently affected their interactions with other mental healthcare providers. Transgender persons responded to cisnormative and hegemonic attitudes by either conforming to the hegemonic expectations in their narratives and gender presentation to receive care or by remaining silent about their concerns and postponing or denying care for themselves. They have committed to this classic trans story in ways that it feels too risky to abandon it to continue to strive for care. This aligns with previous studies on transgender coping mechanisms within public engagement and reinforces the idea of tensions created within transgender persons and how they relay their identities to healthcare professionals.^33–36^

#### Fearing the system

*Fearing the System* (Fig. 1-A3) reflects transgender individuals' positionality in the ways they are viewed by healthcare providers, the manner in which they felt obliged to present their stories to providers, and in fear of the power that providers hold over the course of their lives. For hegemony to function, the conceptions of normativity are directed by the dominant group, affecting cultural norms, education, medical and legal discourses, and so on, in turn creating what Gramsci saw as “exclusion and subalternity.”^32^ Participants consistently downplayed or obfuscated their mental health concerns to their GIATs out of fear that their transition treatments would be delayed or interrupted. They needed to be seen as stable or otherwise risk their transition. Other participants, even when transition was completed, felt that the power imbalance established in their relationships with the GIATs still held sway over how they chose to interact with mental healthcare providers. In these instances, they would downplay or obfuscate their trans identity out of fear that their mental health concerns would be ignored and the only topic of discussion would be them being transgender. This suggests that trans persons did not trust the current system of mental healthcare during transition and experienced a continued distrust of external, or nontransition-related mental healthcare. Poor communication, not listening to concerns, and focusing on “the wrong things” drove these feelings of distrust and fear. This corresponds with the findings of a 2014 study where transgender participants stressed the need to be heard and have their perspectives valued as an essential part of their identity, feelings of self-worth, and basic care needs.^37^

### Foundation of the mirror

The substrate (Fig. 1B) represents the subjectivity of the transgender person through the subcategories of *See Me!*, *Defiant Silence*, and *Wanting Care that Cannot Wait* (Fig. 1-B1 to -B3), where individuals determine their own agenda, reclaim their agency, and attempt to shift the power balance in their favor, even though Gramsci and Spivak might argue that the mechanisms of subalternity still effect the behaviors and attitudes of trans persons in this process. Socially, participants still desired the ability to pass unnoticed as acts of both legibility and visibility and were influenced by the distribution of hegemonic power.^38^ Institutionally, the medicalized view of transition established that permission is needed to be trans. This interweaves into legal and state recognitions and demonstrates the structural powers that maintain dominance. These two elements unavoidably affect how trans persons constructed their own subjectivity.

#### See me!

In the category of *See Me!* (Fig. 1-B1), Spivak's position would assert that the subalterns will always be excluded and cannot be seen in their own terms because of the nature of their marginalization.^39^ Spivak sees this as they are working in a system where they lack representation. While the power disparity is one element, essentially because those providing and deciding care are not themselves transgender, the positionality of trans persons as subalterns could not be changed. It is founded in care plans and filtered down through trans subjects in their navigation of these systems of care. Spivak may contend that so long as transgender persons are at the mercy of the system, the system only works to maintain their subordination. Participants frequently referred to the fact that they wanted to be seen as a person who is trans, not just a trans person. They felt this was not feasible for professionals to understand because they were not trans themselves and their idea of what it means to be trans did not align with actual experiences of being trans.

#### Defiant silence

Spivak would also assert that the subaltern is not an autonomous entity, explaining the idea of *Defiant Silence* (Fig. 1-B2) as an inevitability of subalternization of transgender subjects.^23^ They are not speaking for themselves in their decision to censor or alter their truths in exchange for guarantees of medical treatment. It is the process of being at the mercy of the system and being entrenched in it in ways that recreate, reinscribe, and maintain the subaltern status.^40^ In the instances where participants chose to not speak of their concerns, either about their mental health or being trans, they were still being assessed in ways that reflected the institutional assumptions of gender and of mental health. The fact that they refused to share portions of their lives was born out of a fear of the institutional power held over them. The results from their silence though suggest that their mental health needs often go unchecked and unaddressed. As mentioned previously with regard to *Following the Script*, this coping strategy can hinder interactions with healthcare professionals.^33–36^ Another study on transgender communication strategies noted that this “disengagement” from transgender persons in these relationships often exacerbates the critical issues that need to be addressed and continues a cycle of distress for trans individuals.^41^

#### Wanting care that cannot wait

*Wanting Care That Cannot Wait* (Fig. 1-B3) encompasses an important point made by participants regarding the integration of trans persons into decision-making. This was felt to be vital to remove the subaltern positionality and push back against the hegemonic constrictions that limit mental healthcare for trans individuals. While Spivak would still contend that the position of being subalterns would continue to exist for this group, as they would continue to rely on these structures to obtain care, the prospect of raising transgender voices into the medical discourse creates possibilities to question Gramsci's social adherence to hegemonic norms.^23^ For Gramsci, this change in positionality, with transgender persons directing discussion, could consequently enable them to move from the margins into the center regarding their care.^32^

### Reflective backing of the mirror

It makes sense that two of the elements of the reflection seen from the mirror, *Following the Script* and *Fearing the System*, would also be represented in the reflective coating (Fig. 1C, C1, C2) that helps in creating this image. It shows the influences on behavior that exist for transgender persons. Systemic subordination enables power structures that hold sway over mental healthcare services and cultural assumptions influence the ways in which healthcare providers chose to interact with their transgender patients and how those patients chose to react.

#### Following the script

The subcategory of *Following the Script* (Fig. 1-C1) shows the influence of the hegemonic culture in ways of how transgender persons are expected to perform and relay an acceptable trans story, but from Spivak's perspective, transgender persons do not “speak” for themselves but reiterate back to the structures that keep them subordinate.^25^ Giving the expected responses bolsters the institutions' view of trans subjects and continues a cycle of there being only one way to be trans. Participants remarked that “everyone” knows what they can and cannot say to the GIATs to secure transition treatment. It was recognized that the system views trans experience in a single way and that in presenting themselves as being “in a bad way but not too bad of a way,” they increased their odds of obtaining the transition care they needed but not the mental healthcare they may need.

#### Fearing the system

*Fearing the System* (Fig. 1-C2) frames the authority that places a singular frame of reference on others that adds to the exclusion of the subaltern.^39^ The “system” is the institutional structure that determines the outcome for trans persons in regard to attaining transition treatment. This subsequently influenced their experiences with other forms of care, such as external mental health services, extending the subaltern's entrenchment at the margins of care.^42^ The participants' experiences with care from the GIATs impacted the ways they navigated care beyond transition. Trust and faith in the system were eroded to the point that care was often not sought at all because it seemed pointless. Here Gramsci would describe this reaction as the structure or system ceasing to exist as an external force, and becoming an internal motivator directing hegemonic action.^32^

### Methodological considerations

As the first author (D.B.) is an avowed advocate for transgender issues, familiar with the trans community in general, and has worked with the trans community specifically in Sweden, the cocreating nature of the constructivist approach suited this study well. Participants benefited from the familiarity with trans concerns in a way that added to building trust and rapport that resulted in sharing rich data. To build effectively on this coconstruction, the authors utilized four steps through the analysis process. First, open coding was used to stick close to the words and ideas of the participants to remain grounded in the data.^12,43^ Keeping their reality as a primary frame of reference allowed conceptual analysis to grow organically from their reflections.^15^ Second, extensive, dedicated, and multilevel memoing (substantive, methodological, and analytical) was used to keep focus on the emerging analysis and theoretical formations and integrated into the final analysis. Third, reflexivity notes gave the ability to critically examine influential bias and take steps to minimize it as much as possible. Finally, D.B. used diagramming to aid in connecting texts, codes, memos, concepts, theories and models together.^12^ Throughout all steps, the findings were discussed between the coauthors to ensure that all aspects had been covered sufficiently.^12^

At the same time, the findings also need to be discussed within its limitations. One limitation is the lack of geographical diversity of participants due to limited time and travel resources. Experiences from participants in Göteborg and Stockholm were similar to those in Lund and Malmö, but an opportunity to interview trans persons from each GIAT catchment area may have given other perspectives or other emergent ideas could have taken hold, in particular from participants outside urban centers. This limitation also applied that the recruitment of trans persons as the primary means of advertisement was through specific organizations and shared posts on Facebook. This biased the population of participation to those persons who are members of these groups or who actively seek the services of these organizations or are politically active with them. In previous studies on social media networks and social support systems for trans persons, involvement with these groups was found to be beneficial to health and well-being.^44–46^ Subsequently, individuals who are not part of these support systems may have different experiences with their mental healthcare needs.

While theoretical saturation is the goal, where no new categories can be identified from the data, in reality it is sometimes a point of judgment based on the available resources for the researcher.^12,47^ Similar and overlapping codes emerged from each interview and continued while including probing and clarifying questions throughout the interview sessions. By the 11th interview, no new ideas or concepts emerged and there was a common understanding among the researchers that the views and experiences of transgender persons had been sufficiently covered to answer the study aim. It is, however, acknowledged that inclusion of additional participants could potentially have revealed new information. While qualitative analysis cannot provide the same type of generalizability as quantitative analysis, it provides in-depth knowledge about the experiences and influences among the study population. This study used a relatively small sample of participants, however, as grounded theory does not seek to create generalizability overall, focusing on this specific group and how they related to their mental health experiences, allowed for a deeper understanding for trans persons to emerge.

Regarding the age range in participants, while all revealed similar experiences across stages of transition, it was the two oldest participants (ages 56 and 64 years) who had a noted difference of seeking only informal types of external mental healthcare assistance rather than seeing a psychologist or psychiatrist for their concerns. This could have impacted their perceptions of desired care unrelated to their transition.

Another limitation was using English as the primary language for communication. While every participant was fluent in English, and the introduction of an interpreter would have altered the interview dynamics in other ways, not using the participant's native language inevitably loses some level of meaning or intent during translation. Since all participants utilized healthcare services in the public system, the findings may not be transferable to transgender persons, who use the private healthcare system since there may be conditions and dynamics that are different in this setting. However, it may be noted that on a national level, <40% of primary healthcare services are covered by private practitioners, with an even smaller percentage covering specialist services such as transition.^48^

Finally, as one of the authors is an American, cisgender female and the other a Swedish, cisgender male both associated with a world-renowned university, this brings an associated privilege and positionality of each of these identities into the research process. Despite being advocates for transgender persons, which in itself introduces other limitations in choices made in the analysis, the desire to give voice to trans persons and encompass their viewpoint comes from a detached position of privilege, which invariable limits understanding of their experiences.

## Conclusion

Hegemonic norms support the production and reproduction of knowledge that define gender categories. This in turn grounds policies and procedures for transgender healthcare in these reproduced norms. This study illustrates the ways that these norms serve the system more than the transgender individual. There are numerous ways in which transgender persons are at the mercy of societal norms that erode their autonomy and it is important that medical healthcare professionals work to not add to this erosion. This study intended to examine the ways in which trans persons experience and steer their way through mental healthcare. The results show interconnected factors and influences affecting their processes but ultimately they showed that transgender persons are not getting the care they desire or need. As Sweden is currently taking steps to recategorize the status of being transgender from needing a mental health diagnosis, this momentum should be continued and expanded to include trans persons into the discussions, procedures, and policy formations to free them from their subaltern status within these systems. This will provide a space for this population to begin addressing its mental healthcare to ensure that trans persons enjoy the highest attainable state of mental health as defined by the WHO.

## Abbreviations Used

GIATs: Gender Identity Assessment Teams
M: mean
SD: standard deviation
WHO: World Health Organization

## Appendix 1. Study Information Letter

### Transgender Adults in Sweden: Experiences with Navigating Mental Healthcare Needs

#### Background and purpose

My name is Debra Beight and I am a student in the Master of Public Health program at Lund University (www.med.lu.se/english/public_health). I am writing my thesis on transgender persons in Sweden and I am interested in understanding your perspective on mental health and mental healthcare services. Transgender health issues are discussed regularly, but it is often from an outside perspective. The expertise that shapes policies and practices often overlook the lived experiences of the people who are affected by those policies and practices. There is a lack of research into transgender healthcare that focuses on the needs and expectations of trans individuals, from their own perspective. My thesis is centered on how trans people feel about their own mental healthcare needs and support, however, they define it and whether it is related to transition or not.

I am inviting you to participate in this project because you are 18 years of age or older and identify as transgender or nonbinary. I feel your experiences as a transgender person and your thoughts and feelings regarding mental health and mental healthcare in Sweden are both important and relevant for me to get a deeper understanding of the mental healthcare services that transgender persons receive. Should you choose to participate, your responses will be combined with other interviews and will be analyzed and used exclusively for scientific purposes.

#### What does participation imply?

Participation involves an interview, conducted in English, which is expected to last no >60 min. Please let me know if you would prefer an interpreter. This interview can take place anywhere you prefer and feel comfortable. Using Skype or Google Hang Out to interview online is also an option if you would prefer. This interview will be recorded to transcribe your responses accurately. Your participation is completely voluntary, meaning that you don't have to answer any questions that you don't wish to answer and you can stop the interview at any point and if you no longer wish to participate you can remove yourself as a participant without stating any reason. Interviews will take place between February and March 2017 and you can select a date and time of your convenience. Please note you will be asked to sign a consent form before we conduct the interview. This consent form will be separated from the interview and stored in a locked shelter, only accessible to me and my supervisor, to protect your anonymity.

#### What will happen to the information about you?

All personal information will be treated confidentially and anonymously. During the interview I will be conducting an audio recording but if you don't want to be recorded I can write down what you say instead. The information used from the interviews will be coded so that your name or recognizable descriptions will not be connected to your identity. This anonymity will be maintained throughout the research process and final results, only I will be aware of your identity. The audio recording of your interview will be destroyed on completion of my thesis course in early June.

#### Compensation

I do not have the opportunity to provide compensation for your participation but I am happy to provide fika and refreshment if you wish.

#### Possible risks and possible benefits

There are no known risks or harms associated with participating in this research. Since we will be discussing issues of mental health, if you do feel discomfort about the topic we can take a break, postpone the interview, or stop all together. If you feel it would be helpful to talk with someone after your participation is completed, I can provide you with a list of referrals.

A possible benefit for you would be the opportunity to relay your thoughts, feelings, opinions, and perspectives on the topic of transgender mental health and your relation to mental healthcare here in Sweden. The possible benefits to society would be an improved understanding of healthcare needs for transgender individuals.

If you would like to participate or if you have any questions concerning the project, please contact me at either debra.XXXX @student.lu.se or XXX XXX XXXX. If you would like to contact my supervisor, Dr. Markus Larsson, at Lund University you can do so through mXXXXXX@med.lu.se

Thank you for your time,

Debra Beight

Thesis student

Master in Public Health at Lund University

## Appendix 2. Interview Guide

### Transgender Adults in Sweden: Experiences with Navigating Mental Healthcare Needs

#### Interview guide

Initial briefing: Review the purpose of the interview, sign consent form (if not completed previously), ask for permission to record interview, and ask if there are any questions before beginning.

#### General information

Self-identified gender:

Age:

Transition status: contemplating, started (when), completed (when)

Education: high school, some college, bachelors, masters, doctorate, other (indicate)

Occupation: work, school (indicate)

1. Theme area: general thoughts on mental health
  a. I'd like to start with your thoughts on “mental health”
    i. How would you describe “good mental health” and “poor mental health?”
    ii. When thinking about mental healthcare or support, how would you describe your feelings about it?
      1. What do you think influences your feelings about mental health support?
  b. Statement: “Transgender individuals suffer from higher incidences of mental health illnesses such as depression, anxiety, and suicide attempts.”
    i. Can you share your feelings about this statement?
      1. Can you tell me about some personal concerns you have regarding your mental health?
        a. How might you describe these concerns to a professional (therapist, counselor, psychologist)?
2. Theme area: mental healthcare services related to diagnosis
  a. If transition has not commenced: Can you describe what you think your mental healthcare will be like?
    i. What influences your thoughts on this?
    ii. What do you feel are the benefits to transition-related care?
    iii. What do you feel are the challenges to transition-related care?
      1. How might you handle these kinds of challenges?
  b. If transition is currently underway or has been completed: I am interested to hear what your experiences are with your mental healthcare providers?
    i. Can you describe a typical meeting with one of your providers?
    ii. What are the types of issues you discuss with them?
    iii. What do you think about the services provided to you?
    iv. How would you describe your relationship with your providers?
    v. What are some things you might do/have done differently?
    vi. What would you like your providers to do/had done differently?
  c. Statement: The Swedish Health Board announced in January that it was going to remove the psychiatric diagnosis as a mandatory requirement for transitioning. What are your feelings about that decision?
    i. What does this policy change mean to you?
    ii. How might this change affect you?
3. Theme area: mental healthcare services unrelated to diagnosis
  a. Direct Question: Have you sought out mental healthcare in addition to your transition team? (only ask if it has not been previously brought up)
    i. If no external care has been sought ask question 3b
    ii. If external care has been sought ask question 3c
  b. What do you think are the kinds of experiences that would influence someone to seek out care outside of the transition plan?
    i. Can you describe a time where you felt external care might have been beneficial for you even if you didn't seek it?
    ii. What do you think influenced you to not seek out care?
      1. What might have improved your desire to seek out care?
  c. Would you describe your experiences with your external providers?
    i. What were your reasons for seeking additional care?
    ii. Would you tell me how you went about seeking additional care?
    iii. What stands out as the biggest difference for you compared to your transition team?
      1. How do your discussions differ?
    iv. How, if at all, have your thoughts and feelings about your care changed over time?
4. Theme area: final thoughts
  a. If you could create an ideal mental health support system/plan, what would you want it to look like?
    i. What are the most important elements that would make it ideal?
    ii. What do you think could be challenges in making that a reality?
  b. What is the most important thing you want any mental healthcare provider to know about you?
  c. After having these experiences, what advice would you give to someone who might be in need of mental healthcare?
  d. Is there anything else you would like to share that you feel we haven't covered?

Do you have any questions for me at this time?

Follow-up questions:

What do you mean by…?

Can you tell me more about…?

If we can back up, can we talk about what you said earlier?

Probing questions:

Then what happened?

Can you give me an example?

You mentioned that…?

So you are saying….is that correct or am I misunderstanding?

How do you feel about that now?

Tell me how that made you feel?/How did it make you feel?

Follow-up, probing, interpreting, and subquestions are only as needed should the overarching questions not provide enough thick data.

Debriefing: Inquire if any follow-up or referral is needed and ask if there are any questions.

## Notes

^*^ For the audio/visual communication interview, the participant used an electronic signature for the consent form.
